# Daily practices of advanced practice nurses within a multi-professional primary care practice in Switzerland: a qualitative analysis

**DOI:** 10.1186/s12875-023-01977-y

**Published:** 2023-01-21

**Authors:** Renate Altermatt-von Arb, Hansruedi Stoll, Annette Kindlimann, Dunja Nicca, Elke Lauber, Sandra Staudacher, Monique Sailer Schramm, Franziska Vökt, Franziska Zúñiga

**Affiliations:** 1 Institute of Nursing Science, Department Public Health, University of Basel, Bernoullistrasse 28, 4056 Basel, Switzerland; 2 Spitex Fricktal AG, Münchwilen Aargau, Switzerland; 3 Health Psychologist FSP, in private practice, Zurich, Switzerland; 4 Epidemiology, Biostatistics and Prevention Institute (EBPI), University of Zurich, Zurich, Switzerland; 5 Department of Thoracic Surgery, Bern University Hospital, Bern, Switzerland; 6 Department of Health Services Research, Care and Public Health Research Institute, Maastricht University, 6229 GT Maastricht, The Netherlands; 7 MediZentrum Täuffelen AG, Täuffelen, Switzerland; 8 MediZentrum Schüpfen AG, Schüpfen, Switzerland

**Keywords:** Advanced practice nursing, Primary care, Qualitative research

## Abstract

**Background:**

The rising global population of older persons with chronic conditions demands new primary care models. Advanced practice nurses (APNs) can help meet that need. In Switzerland, APNs have only recently been introduced in primary care and little is known about their daily practice. This study aims to describe APNs’ activities and general roles at four sites with multi-professional primary care practices in the Swiss cantons of Bern and Solothurn.

**Methods:**

To study the practices of APNs at the study sites, we adopted a social constructivist perspective, lending methods from ethnographic field research. We interviewed, observed and accompanied participants over five months, generating rich data on their daily practices. The analysis followed Braun and Clarke’s six-step thematic analysis process.

**Results:**

The APNs’ daily practices cover three main themes. Their core activities are working with expanded clinical skills and being on-site specialists for patients and their relatives. These practices are surrounded by net activities, i.e., taking care of patients in tandem with the physicians and regular visits in residential long-term care facilities. The outer activity layer consists of cohesive activities, with which APNs anchor and facilitate their role and catalyze further development of the care model. APNs tailor their expanded medical knowledge and nursing practice to maximize the value they provide in patient care.

**Conclusions:**

This study extends our knowledge of APNs’ daily practice within a Swiss multi-professional primary care practice. Our results indicate competencies that need to be integrated in APN education and point out the high potential of APN integration in such primary care practices.

**Supplementary Information:**

The online version contains supplementary material available at 10.1186/s12875-023-01977-y.

## Background

The rising population of older people with multiple chronic illnesses demands for new models of care [1]. Globally, two-thirds of health problems result from chronic conditions [2], which generally become more prevalent and add in number with advanced age. In 2017, persons over 60, many of them chronically ill, already accounted for one-fourth of the overall European population [3]. In Switzerland the 20% chronically and/or severely ill health care users account for roughly 80% of health care expenditures [1], in the US, people with chronic and mental health conditions account for 90% of the nation’s annual health care expenditures [4].

Swiss primary care includes preventive and curative services, rehabilitation and palliative care, and is expected to cover the needs of the entire population [5]. However, more than a third of Swiss patients over 65 mentioned in a survey not having easy access to health care professionals, for example, to receive advice [6]. This service gap indicates that the current care model, which is built around acute care, is inadequate, given the increases in chronically ill persons among the older population, the shift from in-patient to ambulatory treatments and a simultaneous shortage in health care personnel [1, 7]. New care models are needed aiming for high-quality, patient-oriented, and economical health care. Guiding principles such as prevention, improved coordination between service providers and increased continuity of care should shape the implementation of such new care models [1].

One promising approach are care models with Advanced Practice Nurses (APNs). The APN designation serves as an umbrella term and includes, among others, (1) clinical nurse specialists (CNSs), who primarily work on hospital wards within interdisciplinary teams, tending to vulnerable in-patient populations and (2) nurse practitioners (NP), who tend to their own patients, providing ambulatory assessments, diagnostic testing and treatment [8]. An APN is a registered nurse who has earned a master’s degree and acquired expert knowledge, complex decision-making skills and the clinical competencies to deliver advanced levels of care [7, 9].

In Australia, Great Britain, Finland, Canada and the US, APNs have been established for decades [10]. In the US, they meet part of the rising demand for primary care services [11]. Successful general practitioner (GP)-APN co-management on the patient level has been shown to heighten patient care quality and increase access to treatment on the level of involved health care workers. In this context, co-management means that two primary care professionals share the responsibility for all tasks needed to manage each involved patient’s health care. Co-management has been shown to reduce the involved clinicians’ individual workloads and prevent burn-out [12]. Therefore, a 2017 position paper from the Organization for Economic Collaboration and Development (OECD) advised member countries to implement new nursing roles in such care models [13]. One potentially useful approach is to optimize the distribution of work and competencies across health care professions [1].

Despite international evidence and recommendation for care models with highly-trained APNs, the Swiss primary health care currently involves few APNs [10, 14]. A secondary descriptive data analysis in an urban Swiss walk-in practice showed that 53% of consultations, involving, for example, non-complicated wounds and upper respiratory tract infections, fall within the range of APN competencies [15]. While qualitative data has shown that Swiss GPs are open to collaboration with APNs, they know little about their advanced competencies [16, 17]. Quantitative data from a Swiss pilot project suggest that APNs are particularly well-suited to handling older, multi-morbid patients with polypharmacy [18].

In Switzerland’s Bernese Seeland, one innovative care model that includes APNs is used by the MediZentrum multi-professional primary care practices in Schüpfen, Täuffelen, Messen and Lyss, where it is the hub of regional care with numerous GPs, counselling physicians, APNs and other care professionals [19]. In fall 2011, the first APN started her work there. APNs’ roles and activities have developed and proven successful in these day-to-day practices: since start, further APNs were hired so that by August 2022, there were two APNs in each of the four MediZentrum locations.

In a collaborative effort between the MediZentren and the Institute of Nursing Science in Basel, this study’s long-term goal is to evaluate the effectiveness of Swiss primary care models with APNs. To do so, we started describing the changes in GPs’ professional roles due to the introduction of APNs [20] and in a next step, the description of the practices of APNs in a multi-professional team.

## Methods

### Study aim and design

The aim of this study was to describe APNs’ activities and general roles [21]. A qualitative approach was used to gain understanding of everyday APN practice. Methodologically we used a social constructivist orientation with ethnographic elements of field research. These included individual interviews to understand the APNs’ subjective perspectives and a “go-along” approach, where the first author (RA) accompanies the participant according to Kusenbach to gain understanding on habitual processes [22]. One basic premise of social constructivist theory is that people construct the world in which they live and work through their culture and language. The “go-along” approach is a type of participant observation using a “systematic and outcome-oriented version of ‘hanging out’ with key informants” [23, p. 154]. One strength of the “go-along” approach is that, through a combination of open-ended and semi-structured interviews during participant observation, it is possible to both listen to what APNs tell about their daily practice and see them doing it [23]. All data were analyzed following Braun and Clarke’s approach to thematic analysis, which allows insights into day-to-day activities to describe the meaning of ANPs’ practices, highlighting commonalities and differences that would not otherwise be obvious [24, 25].

### Research setting

Each of the four Berner Seeland MediZentrum locations is a multi-professional primary care practice, the services of which include home calls and calls in residential long-term care. Each is an independent corporation with several physicians on its board of directors. Each practice employs four to seven GPs, APNs, medical practice assistants and coordinators, medical secretaries plus gives space for consultations for other health care professionals including consulting specialists like a cardiologist or a rheumatologist, psychologists, diabetes and nutrition experts. The team cares for patients of all ages for injuries and acute and chronic illnesses [26–29].

### Study sample and procedures

The research group aimed to include all four APNs working in the four Bernese Seeland MediZentrum practices at the beginning of data collection (August 2019). The local coordinator, who approached the university for the overall study, recruited all participating APNs personally or via email. All four were given the opportunity to discuss questions with the first author and have provided written informed consent. The local coordinator (MSS) is also a co-author of this study and one of the participating APNs. She did not participate in the data analysis and interpretation except for the member check. In May 2019, the first author informed the APNs about the study, gave them the opportunity to make adaptations regarding the proposed manner of data collection and answered their questions. In this first contact, she also transparently communicated her positions as an APN at the University Hospital Basel and as a master student in nursing science. During the conceptual phase, feasibility-related and contextual factors for interviews and go-alongs were clarified through regular contact with the local coordinator.

### Data collection

Data were generated in the APNs’ various practice locations (residential long-term care facilities, patients’ homes, MediZentrum practices, etc.) and included interviews and go-alongs. The first author collected data over a five-month period (August 30th, 2019 to January 9th, 2020). The initial interview with each APN focused on daily practices (duration mean 1 h 21 min) at their work locations. These interviews started with the following three sentences:

*So far, we know little about the everyday work of an APN in Swiss primary care. With this study we want to understand what you do as an APN and what your day-to-day practices entail. What comes spontaneously to mind?*

The individual interviews also included the collection of demographic data (age, gender, education and training, professional experience, current position). Every interview was followed by two go-alongs, which were conducted according to Kusenbach [30], each lasting 3–5 h on separate workdays. During these eight go-alongs, the first author accompanied each APN through her daily activities, participating in their day-to-day operations [31] and using reflective observation, informal conversations and listening to gain access to their activities and experiences [30]. The semi-structured protocol for these was based on the research question and included segments on structure, persons and processes [32]. In line with the research question, the go-alongs included observation of the APNs’ daily practice and activities, how the observers experienced them, and the context of each within the relevant MediZentrum practice. Twice during the go-alongs, apart from informal conversations, 30-minute exchanges took place regarding the APNs’ workdays. Both included clarifying questions and background information about the APNs’ daily practices.

Whenever possible, potentially involved persons, such as patients and persons with whom the APN would come into contact on the days of the go-alongs, were informed beforehand by means of information leaflets (e.g., leaflets were distributed in the nursing homes where the APNs worked). Within the scope of the go-alongs, as not all interactions could be foreseen, it was not possible to provide advance notice in the traditional manner to persons involved coincidentally. Other measures would have severely interrupted natural process of social interaction. Considering the study’s focus was on the APN and an ethical reflected manner on the part of the first author, consent was negotiated either through non-verbal interaction (based on reactions to the presence of the first author) or verbally via a brief explanation [22].

One to four weeks after the latest go-along with one APN, via a telephone interview (duration mean 30 min) the first author reflected and supplemented her collected information and observations. The first author digitally audio-recorded all interviews, supplementing the recordings with field notes. The guidelines for the interviews and the semi-structured go-along protocol were adjusted based on the findings. For each APN, four face-to-face and four telephone interviews were transcribed in standard German. Overall, the first author transcribed 33 h of interviews and observations from go-alongs into a detailed record. Elicited names and places within the data were pseudonymized.

### Data analysis

Demographic data were presented descriptively. Further analysis of data was inductive, based on thematic analysis (TA) according to Braun, Clarke, Hayfield and Terry [33], supported by MAXQDA software (release 18.2.3). We used an inductive approach to develop a codebook with different themes and subthemes. For this, the first author encoded the data based on the research question and produced mind maps. The research group moved through four phases: “familiarizing oneself with the data,” “construction of the subject,” “subject review” and “definition and theme designation.” The identified themes were drafted as a code tree and served to capture the essence and patterns of the data (see supplementary file 1). In April 2020, assisted by a fellow student (EL), the first author led all four studied APNs through a two-hour member check to validate the drafted code tree. This member check was digitally audio- and video-recorded, then summarized in note form. It was also depicted as a knowledge map during the meeting. Based on the feedback and via extensive analytical work, the research group developed a codebook and a three-themes conceptual model. They agreed that redundant data were present. This indicates that all existing themes had been incorporated. The first author wrote the report.

### Ethical considerations

The small size of the MediZentrum interdisciplinary team makes full anonymization of the data because of context factors impossible. Therefore, before this report’s finalization, the participants could request adaptations regarding confidentiality. The clarification of jurisdiction with the Bern cantonal ethics commission brought an assurance that this study did not require their ethical approval (BASEC-Nr.:Req-2019-00513).

### Strategies to ensure reliability of results

Over the course of this study, data were discussed repeatedly in peer-groups at the University of Basel’s Institute for Nursing Science, within the core research group with two experienced researchers (FZ, AK) and two master’s students (EL, RA), and with both first and second thesis supervisors (FZ and HS). Regarding quality assurance, the analytical processes were recorded comprehensibly. Via member checks, the studied APNs validated the data, confirming that the code tree reflected their daily routines.

## Results

### Participants

At the time of the interviews, the four APNs were between 26 and 47 years of age, and had three, four, sixteen and 23 years of professional experience as nurses. All four fulfilled the criteria of an APN according to the consensus of the Swiss universities and colleges: a master’s degree with a minimum of 90 ECTS (1 ECTS includes 30 h study time) and a focus on advanced practice nursing [34]. Two had also earned additional diplomas: one in advanced studies in management for health professionals, the other a further specialization in APN training. They had worked between one and eight years for the MediZentrum Bernese Seeland practices, and were employed from 3 to 4.5 workdays per week. Before joining the MediZentrum workforce, all four had worked in hospitals in various professional disciplines. In addition to her hospital position, one worked as a community nurse and was self-employed. Our results amalgamate all four APNs’ data.

### Activities of APNs

**Fig. 1:**
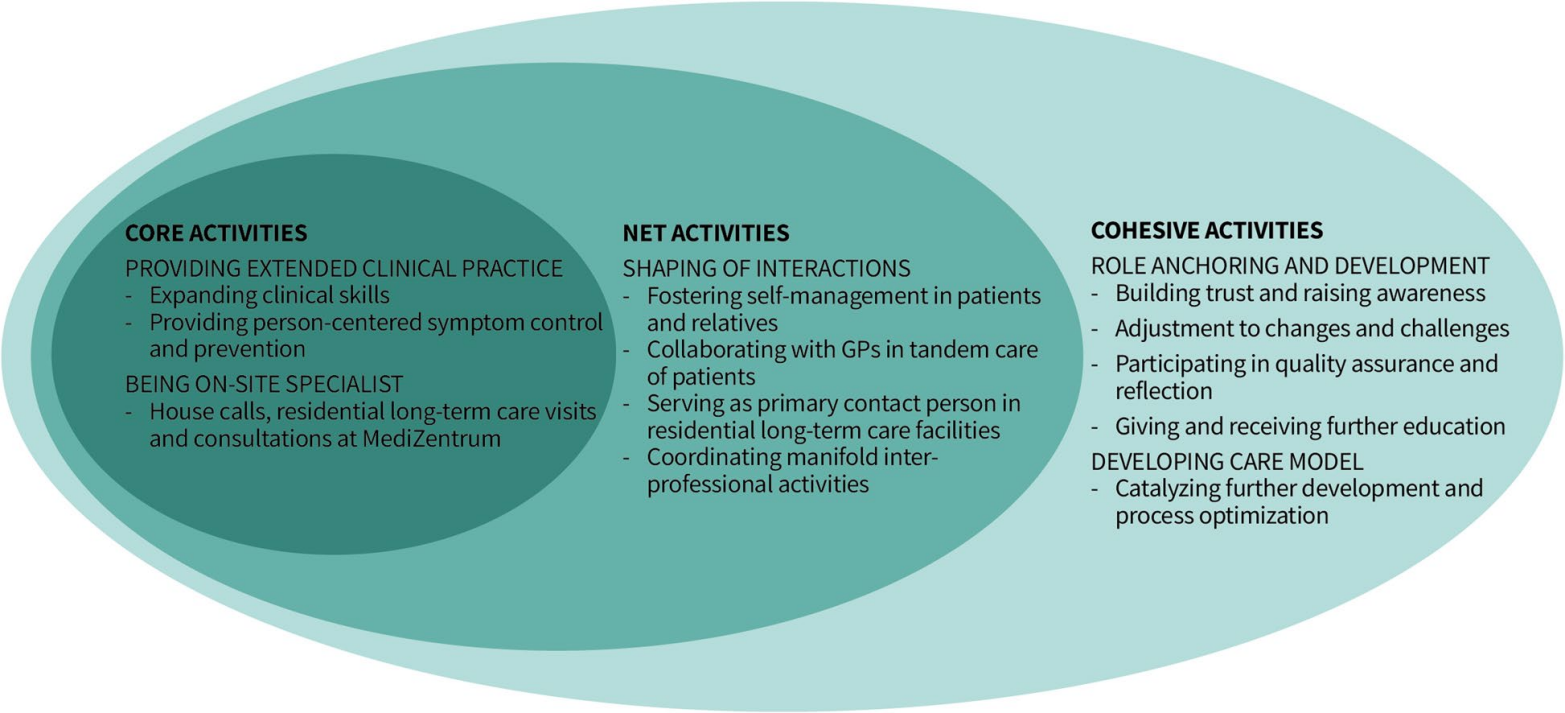
Everyday APN practices in a multi-professional primary care practice

With the core activities in the inner circle (cf. Fig. 1), we describe the hands-on activities of APNs when they deliver extended clinical practice and are on-site specialists. The net activities consist of shaping the interactions needed to provide the core activities, be it with patients and their relatives, the GPs, with which they work in tandem, or other health care providers. While the APNs use their cohesive activities to anchor their roles in their care settings, they also continuously develop those roles as well as their overarching care model. This impacts both their core activities (clinical practice) and their interactions in net activities.

### Core activities

The core activities describe the daily hands-on activities of the APNs, combining their nursing background with a daily routine approach to symptom control and prevention, while expanding their clinical skills. Although patients occasionally visited the MediZentrum practices, for these core activities the APNs in our study usually visited patients on-site in their homes or in the residential long-term care facilities.

#### Providing extended clinical practice

##### Expanding clinical skills

Many hands-on activities we observed in the go-alongs refer to APNs’ clinical skills building on a needs-based assessment. The patients APNs were responsible for, are mostly chronically ill, multimorbid and geriatric. According to the go-alongs and interviews, the APNs started with the anamneses covering biological, psychosocial, cultural, spiritual and daily routine perspectives, bringing together the nursing background and additional medical skills they honed under the supervision of the GPs. Based on the anamneses, the APN conducted a needs-based physical patient status assessment. The APN also identified where laboratory analyses were necessary, took the appropriate samples (normally blood or urine) and decided together with the GPs which tests to run. The APNs then interpreted and prioritized the collected data and initiated appropriate nursing measures, taking into account each patient’s overall personal situation, which often required conversations with relatives. The APNs independently initiated, monitored, evaluated and adjusted the therapies within both their extended health care spectrum and the limits set by their supervising GPs (e.g., antibiotics are always ordered by physician, see case vignettes, Table 1). All medical decisions were documented and discussed with and confirmed by the treating GP to ensure that APNs were acting within their defined limits.

APNs’ autonomous clinical practice could be observed in numerous cases during the go-alongs, e.g., intervened in cases of excretion difficulty, performed ear irrigations. Where necessary, the APNs adjusted patients’ medications within the agreed-upon limits given by GPs, e.g., anticoagulants or diuretics, gave pain management advice. In situations where an APN saw a case for either reducing a dosage or discontinuing a medication, she discussed it with the treating GPs and adapted accordingly. Moreover, GPs taught them manual skills, including residual urine determination or Cystofix catheter changes. In the interviews, the APNs also reported providing palliative support, initiating hospitalization, or administering intravenous immunoglobulin with GP support in the background.

##### Providing person-centered symptom control and prevention

The APNs’ person-centered symptom control and prevention methods were tailored to the patients’ anamnestic, clinical and laboratory results. They began by determining symptoms and resources (see case vignette Table 1), then recorded symptoms based on the physical status. In many cases, the APNs already knew the individual background and environment of the patient, e.g., which relative was responsible for which care aspects or which topics bring motivation and joy to the person. Accordingly, they were in a position to tailor the symptom control to the person. Once yearly, they also conducted a thorough evaluation of all relevant laboratory and vital values for each patient under their care. This helped them prevent, e.g., dyspnea resulting from anemia, or weight loss and mental depression caused by thyroid dysfunction. Likewise, the APNs systematically evaluated each patient’s list of diagnoses and their levels of escalation. These levels of escalation contain decisions regarding resuscitation, hospitalization, intensive care and antibiotic administration.

**Table 1:** Case vignettes: *providing extended clinical practice*

**Expanding clinical skills**
Mrs M. is an 87-year-old residential long-term care resident with chronic heart failure (NYHA IV), restrictive pneumopathy receiving long-term oxygen therapy. She has a low BMI and is developing dementia. After a stay in hospital due to cardiac decompensation, the APN visits her at the residential long-term care facility. Following a need-oriented anamnesis, the APN conducts cardiac and pulmonary checks, including auscultation, as well as an assessment of the patient's legs and neck veins and a survey of vital parameters. She checks her adjusted oxygen and educates Mrs M. regarding the supplementary drinks concerning indication, who takes over the costs, as well as how and when to take them. The evaluation of the patient's most recent hospital admission is as important as the APN’s active responsiveness to social narratives and question as well as information Mrs. M. wants to pass on to her GP. The APN concludes that the current medication and dosage should be maintained. While saying goodbye the APN ensures their next contact point. (APN_4, go-along)
The networked way of thinking (is) what is needed. [..] At the hospital you have delegated work processes. And here is the point where you have to start to network yourself; you have to think a step ahead. When you visit to draw blood. [..] Someone coughs; you go to auscultate. [..] Then you decide, I draw blood. You have to consider, what am I going to have examined in the sample? [..] Sometimes they receive antibiotics. [..] Maybe one should discern the kidney levels. [..] Afterwards I visit the GP and report about the situation, how the lungs sound, the vital signs, the laboratory levels are such. Then he basically just has to say, ok, yes, we have to administer antibiotics; and therefore, he prescribes them. (APN_2, interview)
**Providing person-centered symptom control and prevention**
An estimated 90-year-old resident with dementia has recurrent urinary tract infection. During these infections, she has visual hallucinations of snakes. Today, while visiting to evaluate the patient's blood coagulation, the APN also uses the meeting to inquire about the snakes to evaluate the presence of an infection. The resident reports that they are currently not there. (APN_4, go-along)

#### Being an on-site specialist

##### House calls, residential long-term care visits and consultations at MediZentrum

Within the MediZentrum treatment team, the APN is responsible for ensuring the long-term stability of the patients assigned to her. For patients and relatives, she is the on-site specialist and contact person providing continuity, whether in the scope of house calls, facilities for the disabled, in residential long-term care facilities or in a wound or diabetes consultation at the MediZentrum. House and residential long-term care visits offer the advantage that mobility-limited and cognitively impaired patients can stay on-site while the APNs can make an assessment of their living area (see case vignette Table 2). This extra information sharpens their focus on allowing patients the best possible life with their conditions, ensuring that therapeutic decisions are compatible with the living context of patients and the care givers involved.

Each APN acts as a bridge between a GP and a patient. For example, they could make quick adjustments immediately in the patient’s home. The APNs in our study noted that their patients appreciated the regular, reliable presence of a professional like the APN in their proximity as well as a nearby access to necessary therapies, e.g., intravenous immunoglobulin treatments (see case vignette Table 2), without going to a far distanced hospital.

From the APNs’ perspective, house calls relieve the burden of relatives, as they allow proactive coordination with the patients and their relatives to minimize the burdens both of illness and of therapy on the patients’ daily lives. The APNs also perceived an improved sense of security in patients through their proactive approach and on-site presence. Further, although data were not available on this effect, the APNs perceived a reduction in telephone calls and visits to the MediZentrum.

**Table 2:** Case vignette: *Being an on-site specialist*

**House calls, residential long-term care visits and consultations at MediZentrum**
During rounds at a residential long-term care facility, a GP calls the APN to share the background concerning a patient who urgently needs a home visit that afternoon. The geriatric patient has been experiencing reduced overall health for the last week, has signs of a cold and drinks nearly nothing. The patient was seen last week by the same GP. Having worked with the patient and her family for some time, the APN knows that in this multi-generational household the son and his wife normally take care of the patient. At the practice the APN and the GP discuss the case. During the visit the APN speaks to the patient and the daughter-in-law about a possible course of action. Following the GP's instructions, as the patient is unable to swallow pills, the APN brings an antibiotic suspension. While administering a 0.9% intravenous NaCl infusion she documents the added information to the anamneses and the interventions. The APN informs the patient that she might soon need to use the toilet several times, as she has received fluids. Although the toilet is not far away, the patient is weak at the moment and could ring for assistance from her daughter-in-law anytime. With the patient and daughter-in-law, the APN arranges for a timely follow-up visit. (APN_1, go-along)
They [the residential long-term care residents] appreciate it [blood transfusions at the MediZentrum practice] tremendously; and they are more willing to accept such a measure to diminish symptoms and less likely to just want to die quickly because everything is so bad. (APN_4, interview)

### Net activities

To ensure the extended clinical practice, the APNs foster interactions with diverse partners. Interactions occur, among others, when fostering self-management of patients and their relatives, in the collaborative care of patients with GPs, during rounds and care coordination efforts. These interactions constitute the net that holds their many tasks together.

#### Shaping of interactions

##### Fostering self-management in patients and relatives

One of the APNs’ strategies to improve patients’ and their relatives’ self-management skills is to increase their understanding of the relevant illness and therapy and their implications for managing their everyday lives (see case vignettes Table 3).

Psychosocial themes the APNs discussed range from support in decisions regarding living arrangements, or how they have been feeling since their partner’s passing away to who takes care of their cat during hospitalization. Often in close interaction with relatives, the APN consciously conveyed appreciation. Also, the APN ensured the flow of relevant information between the family members, even if they are not present.

##### Collaborating with GPs in tandem care of patients

Working in tandem means jointly defining a treatment goal, determining each work order clearly and exchanging information should questions arise about the patient’s situation. One APN had fixed consultation meetings with her collaborating physicians; two are in the process of implementing these; and one worked mainly via written exchanges. Consultation appointments around the lunch break have proven effective. The central point for APNs was that discussions and written exchanges happen. At the same time, there was variation in the amount of information desired by the GP, which duties were delegated and how teaching and supervision were shaped. During the meetings, the APN related her current clinical estimation. Limits for medication adjustments are defined, intervals and manners of control are set and, with attention to the patient’s preferences, therapy procedures defined (see case vignette Table 3). The APN perceived these collective decisions as a relief to both parties.

##### Serving as primary contact person in residential long-term care facilities

During the admission process for residential long-term care patients, either the APN or the GP led the discussion about the care plan. Based on the history and examinations, they discussed the care plan and escalation levels with the patient, a family member and a nurse from the facility, including the patient’s vision on treatment goals and wishes concerning hospitalizations, resuscitation, and antibiotics. This exchange is also used to introduce the APN’s role as primary contact person from the MediZentrum, who will then follow up based on the goals established. After the admission process, APN and GP went on rounds together once a year, unless there were new or acute situations.

As the APNs are MediZentrum’s primary contact persons regarding the residential long-term care residents’ concerns, they conducted regular, usually weekly, visits on site (see care vignettes Table 3). These regular rounds enabled proactive treatment. One or more reliable and competent nurses per residential long-term care facility are essential for an efficient and productive collaboration with the APN. Typical activities on visits were symptom monitoring, medication dosage adjustment, consultation, examination, or onsite blood sample testing. Underlining that “communication on eye level” is key, the APNs often asked for the nurses’ and care workers’ opinions. If needed, they also provided coaching and background information on medication and the handling of medication on demand, and supported the assessment and handling of challenging situations.

##### Coordinating manifold interprofessional activities

Depending on the patients’ situation, the APNs coordinated the work with legal assistance, specialists, physiotherapists and social service staff. They coach medical practice assistants and coordinators, and were responsible for follow-up on practical recommendations.

The gap of unavailable (hospital discharge) reports limits coordination quality, e.g., follow-up appointment. The interactions with home care personnel range from wound experts to case managers. They are also requested to conduct roundtable discussions with involved professionals and care receivers. Through close interaction with relevant actors, APN proactively obtain valuable knowledge regarding their patients’ situations.

**Table 3:** Case vignettes: *Shaping of interactions*

**Fostering self-management in patients and relatives**
I had a patient [...] who suffered from severe heart failure. [...] She had weighed herself, measured her own blood pressure and always recorded it. [...] One time she had received intravenous diuretic from her home care nurses in consultation with me. After that I told her, ‘We have to check this when you report it.’ [...] I then asked her, ‘When did you first notice the breathlessness?’ or ‘What made you notice this?’ [...] With questions I found out that, aided by a rollator, she walked the same short walk every day - and that she suddenly noticed that that short walk did not go as well anymore. Then I said ‘Now we have it. If you notice that this short walk does not go as well, then you have to call me so I can come by. Even if it is a week earlier [than our next planned appointment].’ And that worked. (APN_2, interview)
When a patient independently reached out to the APN after a hospital release, of which the APN had not been informed, the APN described it as a success. (APN_1, interview)
**Collaborating with GPs in tandem care of patients**
The condition of an 85 year-old residential long-term care resident with decompensated heart failure and elevated inflammation parameters worsened clinically with antibiotic treatment and intravenous diuretic infusions. After consultation with the treating GP, the APN talked to the patient and family members, on-site at the residential long-term care facility, about the goal. Treatment focusing on stabilizing the patient's quality of life allowed a regular hospital admission the following day rather than an emergency admission that evening. In the meantime, the long-term care resident is back in the residential long-term care. She isn’t dyspnoeic and the oedema was gone. (APN_3, interview)
**Serving as primary contact person in residential long-term care facilities**
In the care facilities I have fixed visits. There I know I will be present [on these two days] from 10am. [When I arrive,] the nurses ask me any questions they have or tell me who I have to see. [I] also have ongoing cases; so I say, this one I want to see, this I want to check, because I know the importance of continuity. (APN_4, interview) Visit at Residential long-term care facility V: Ms U (Nurse): Mr W has blood in his urine. After a few specific inquiries the APN asks Ms U, "Please run a Combur [urine] test and let me know the results." She notes the test in Mr W's file in the facility's care folder. APN: On my last visit we reduced Mrs X’s Meto-zerok (anti-hypertensive medication). Ms U: Now you surely want to see the results. They are very good. Neither the pulse nor the overall condition have changed. APN (reading the results): From now on pulse control once a week is sufficient. Ms U: Thank you. Ms U: We have newly a married couple together here, the Y's. Ms Y suffers from severe dementia; her husband has a calming effect on her. Both would like to return home. APN: Mr Y was previously hospitalized because of gastrointestinal complaints. Because of Ms Y's dementia she would be cared for better on a special care unit. They want to recover here and go back home. We can evaluate their progress as we go along. APN: How is Ms Z's erysipelas looking? Ms U: better. The two decide afterwards to visit Ms Z together. (APN_1, go-along)

### Cohesive activities

Surrounding the core of extended clinical practice and the interactions in the net activities, the APNs not only develop and anchor their roles but also develop their care model. Throughout the observation period, it became clear how much concerted effort had already gone into building the APN role and the APN team. Nowadays, the APNs are further developing and elucidating their role and building trust. The APN continuously meet the challenge of anchoring and developing her role, supported by patient conferences, quality circles and continued training. Moreover, the APN is a catalyst of the care model’s development.

#### Role anchoring and development

##### Building trust and raising awareness

Fundamental for the role anchoring is to build trust through raising awareness of the role’s content and designation, and competencies. At the beginning of all collaboration - with patients, within the care team or at a political level – there is building of trust (see case vignette Table 4). The APNs emphasized that one important basis of trust and acceptance among GPs was the experience of their high levels of clinical competencies, both in nursing tasks and in those that overlap with physicians’ roles.

##### Adjustment to changes and challenges

The duties and responsibilities of the APNs were continuously adjusted to their collaborators’ level of readiness to accept and work with them. Moreover, the APNs’ introduction also implied changes in the roles of their collaborators [20]. The APNs reported on one side strong collaborations and on the other side challenges in collaborations.

APNs for example explained that two challenges were residential long-term care managers’ reluctance to collaborate with APNs, and their skepticism towards the APN care model. The skepticism was palpable after a change in residential long-term care management or an exit of key MediZentrum personnel. Changes led to frequent role re-anchoring and re-development. Therefore, the APNs stated it was necessary to have a strong sense of self-responsibility and motivation.

Similarly, a sense of achievement is essential. APNs experience this often in their interactions with patients and based on positive feedback from their collaborators. A sense of unity at the MediZentrum is a key factor that helps the APNs to feel that they fit into the care model provided. For this purpose, each incoming GP is assigned to one APN for in-service training. This gives the APNs opportunities to show and explain their roles. Collaborating with the in-coming physicians at an early stage and deciding with them how to work together fosters early acceptance of these nurses’ roles and competencies.

##### Participating in quality assurance and reflection

Two meeting formats are consistently used to develop and anchor APN roles. The first involves monthly conferences of physicians and APNs. Rotating through the four MediZentrum practices, these begin with the presentation of case examples, which are then reviewed regarding relevant aspects of collaboration (see case vignettes Table 4). Their goal is to promote APNs’ step-by-step implementation in all applicable areas. The second format is the monthly quality circle, in which APNs discuss subjects such as the alignment of processes, ongoing projects and matters of everyday practice. Both formats contribute to ‘Catalyzing further development and process optimization’, as described below. Both are led by the same APN.

##### Giving and receiving further education

In addition to the first meeting format described above, MediZentrum GPs and APNs also periodically present and discuss case examples for regional GP training purposes. Topics include necessary structural adjustments (e.g., regarding health care records), legal anchoring (e.g., physicians‘ legal responsibilities regarding delegation) and compensation for services by health insurers (see case vignette Table 4). Another example of further education involved a commitment within a long-term residential geriatric care facility to reactivate care workers’ knowledge and skills to administer high enemas according to their competencies. The APNs also participate in interprofessional congresses and media contacts, network with expert panels, publications and further their competencies in an interdisciplinary environment.

**Table 4:** Case vignettes: *Role anchoring and development*

**Building trust and raising awareness**
I wish for conversations, persuasive efforts and positioning, for instance concerning the nurses at residential long-term care facilities, they will become fewer in number, that it will suddenly become self-evident that we exist and what our role is. (APN_3, interview)
With certain [patients] I don’t know if it is that present or evident that I am actually performing the role of APN. [...] So even though I explain at the beginning and the GP introduces me as such - and I keep saying it - to some I am the medical practice assistant; to others I am an GP, and this needs repeated correction. And afterwards I realize, they don’t actually care what my title is or what kind of education I have. To them what matters is what I do for them, and if they have the confidence in me that I will take care of it. (APN_3, interview)
**Participating in quality assurance and reflection**
[In patient conferences,] GPs learn, how can I collaborate with an APN? Which duties can I delegate? How are controls to be implemented? How can we divide these job groups, so [it] is safe from an implementation perspective, so that the GPs can bear the responsibility (which they still have), so that everything is all right for patients and their family members? (APN_4, interview)
GP during a patient conference: 'The APN has the focus on the resident, which is completely right. I don’t have to be on-site to make the diagnosis. It is either an infarct or a hemorrhage. But to the resident and the family members it is important for someone to be on-site. A process has been initiated. The family can now take a step, if they wish to do so. As an APN she is perfectly suited for such situations. [...] the GP would be under much more time pressure.' (APN_3, go-along)
**Giving and receiving further education**
[It] is a little bit my hope that somehow, based on the Seeland example, the APN role in the primary health care system can also be transferred. [...] I believe the basic idea that the way we practice here is surely something that people can profit from everywhere. And it would be lovely if it could happen this way someday, that [this care model] would be self-evident. (APN_3, interview)

#### Developing care model

##### Catalyzing further development and process optimization

The APNs noted a shift in their activities away from in-practice consultations towards house calls and residential long-term care services. One experienced APN is proactively working within two interprofessional work groups to further develop the MediZentrum care model. She was coordinating an evidence-based cross-center diabetes and colorectal treatment plan with the aim of providing uniform care quality.

## Discussion

This study of APNs employed by MediZentrum’s four Bernese Seeland practices shows that these nurses’ everyday work contains three main scopes: core activities, net activities and cohesive activities. At the core of their daily routine are the provision first of extended clinical services—combining both traditional nursing and GP tasks—and second of on-site nursing specialist knowledge. These core activities are held together by the APN’s net interactions. All around, cohesive activities help both to anchor the APNs’ roles and to develop both those roles and the care model. We show the adaptive practice of APNs in a multi-professional primary care practice and the needed central elements. In the MediZentrum’s practices, APNs add tremendous value by expanding their extended classical nursing competencies to include what have traditionally been considered medical skills. Our results indicate a strong potential for APN use in primary medical care in Switzerland.

### Core activities

For several decades APNs have cared for housebound older people. Managing these patients’ health and medical conditions is one of their major care areas [35]. Alongside the importance of care continuity, our results reflect the central theme of making the best out of the patients’ health and medical conditions. In Belgium the most suitable tasks for APNs were found to be patient education and technical nursing skills [36]. While we observed APNs perform different clinical skills, they were always used in view of a patient-centered symptom management and to support patients and their relatives in dealing with their diseases in their current context. This differs from Guillaumie et al.’s 2019 observation that APNs in Canadian primary medical care felt pressured to practice according to a biomedical model, constantly having to defend their role of supporting self-management and feeling conflicted to compensate for the lack of GPs [37]. In the MediZentrum practices, the APNs’ tasks clearly complement those of the GPs, with no mention of pressure to conform to a biomedical model. On the contrary, in this practice, each profession was able to bring its strengths to the patients’ needs.

In the coming decade, ensuring the continuation of high-quality primary care will require a great number of trained professionals. Care models with APNs can help meet those needs [38]. The present description can also provide the basis of a clear profile for APN roles in Swiss primary medical care, much of which is delivered to patients in residential long-term care: Given that many patients keep their GPs when they move into long-term residential geriatric care homes, primary care also covers this setting.

### Net activities

Self-management support of chronically ill people is a common activity for APNs [34, 37]. Our study results indicate that high interaction-related competencies are central to APN roles, both in contact with patients and their families and with other health care professionals. In Canada, APNs in primary medical care spoke of promoting interprofessional communication skills, noting a particular need for mentoring from GPs who are dedicated and have time for it [37]. Meanwhile, in the US, GPs and APNs have recognized a mutual need for close collaboration [39]. For functional co-management, GPs and APNs alike identified effective communication, mutual respect and trust as key factors for success [12]. And in Switzerland, recent findings have linked increased exchanges with stronger GP-APN collaborations [40]. The current study’s qualitative insights confirm that, for APNs, regular, need-adjusted interactions with their collaborating GPs are essential.

### Cohesive activities

Developing and anchoring APN roles require enormous time and effort. Part of the challenge is to recognize, specify and communicate the ‘nursing’ part of the APN role. APNs’ added value lies within their expertise regarding specific conditions and therapies and their effects on patients’ and their family members’ daily life—particularly regarding activities of daily life. This includes seeing firsthand what these patients and their family care givers need to manage—as opposed to what they actually *can* manage. House calls and residential long-term care visits are key components. APNs’ unique combination of advanced nursing and medical abilities makes them an ideal choice for such visits.

Among care teams, those that implement institutional processes to support clear role division tend to yield the best outcomes [40]. Alongside team functionality, clear role definition is the most common issue, as it influences interprofessional collaboration [41]. To ensure role clarity in daily operations, MediZentrum has two proven formats: patient conferences and quality circles. The literature shows that formats which foster a clear understanding of the different roles are essential [40]. One further approach to role anchoring - which is also expandable - is to assign part of each incoming GP’s in-service training to an APN. The implementation of both quality assurance formats and the use of APNs to orient physicians suggest high effectiveness of MediZentrum’s interdisciplinary services. One effective national strategy to assure high-quality care is to register with APN-CH, the association responsible for quality oversight in advanced practice nursing. This option has been available to APNs in Switzerland since March 2021. The registered title’s “CH” suffix indicates Switzerland (“*Confoederatio Helvetica*”) [42].

### New health care model

By incorporating APNs in their daily primary care processes, the medical team from MediZentrum practices built a new care model. The goal of new care models is to provide high quality, patient-oriented care that is also efficient and economical [1]. Based on the APNs’ everyday practices in this study, the MediZentrum care model already covers several recommended aspects of new care models like optimized access to care through proactive residential long-term care visits (see Table 5). This indicates that including APNs in primary health care models would provide manifold benefits.

**Table 5:** *Central ideas for new health care models*

Central ideas for new health care models [1]	Results of the current study
1) Prevention und empowerment	• Providing person-centered symptom control and prevention • Fostering self-management in patients and relatives • Supporting patients and family care givers with effects of illness and therapy on everyday life
2) Optimized access to care	• House calls, residential long-term care visits and consultations at MediZentrum
3) Treatment guidelines	• Catalyzing further development and process optimization • Expanding clinical skills • Collaborating with GPs in tandem care of patients
4) Improved integration and coordination	• Coordinating manifold interprofessional activities • Serving as primary contact person in residential long-term care facilities • Collaborating with GPs in tandem care of patients
5) Heightened commitment and continuity	• Being on-site specialist: House calls, residential long-term care visits and consultations at MediZentrum
6) Patient-oriented care and self-management	• Fostering self-management in patients and family care givers, adjusted to their abilities
7) Improved quality and patient safety	• Participating in quality assurance and reflection • Giving and receiving further education

### Method discussion

To be able to judge the transferability of our findings, we focused on producing full descriptions of the contexts and results. Regarding our data’s credibility, they accurately reflect the participants’ points of view. Within our thematic analysis, as we interpreted our own data, the criteria did not include confirmation [43]. Considering that subjective points of view, an external view and real work days complemented one another well, the combination of interviews, go-alongs and a member check suited our needs. The insight and reflection this allowed made habitual actions visible and allowed answers to our research question. The participating APNs’ daily practices were observed by a person with a professional nursing background. This study has explored in depth only one Swiss multi-professional primary care practice. Therefore, to transfer this care model to another context, it will be necessary to acknowledge and analyze the new context and adjust this model accordingly.

### Recommendations for practice, teaching and research

#### Practice

This qualitative examination’s results suggest that the Swiss primary health care system would benefit from care models which include APNs. They can essentially contribute to primary health care by combining classic nursing skills with essential medical tasks. The authors recommend to work on a concise description of the APN roles. Our findings suggest that a specific role profile for APNs in the primary care system would improve current practice. To further expand the full range of APNs’ core, net and cohesive activities, we recommend that the new care model embed the supportive structures observed here, including regular consultations with GPs, regular visits at residential long-term care facilities, patient conferences and quality circles. Regarding APN compensation, as a starting point, we recommend shifting away from physician-billed fees to a blended team- and performance-based system [13]. The authors underline the importance of primary care APN registrations.

#### Teaching

The three areas of practice observed here provide evidence that Switzerland’s APN education should allow increased specialization. For example, in Canada, the US and Spain, APNs can specialize in primary medical care [44–46]. In the US, 85% of nurse practitioners’ graduate training prepares them for work in primary care [47]. In Switzerland, though, their implementation in primary care is limited to a small number of pilot projects [17].

Still, APNs’ value in primary health care indicates a need for general practice-focused training at the university level. In particular, to accompany symptom control and prevention in older and chronically ill persons, a focus on clinical techniques is recommended. The importance of such knowledge has been demonstrated in the literature [48]. Besides the expansion of manual skills (e.g., ear irrigation, determination of urinary retention, Cystofix catheter changes), instruction should focus on supporting patients’ and relatives’ self-management capacities to help them cope with everyday life. To foster a clear image of APNs’ responsibilities, course content should include tasks on developing and anchoring roles, building mature communication skills [37] and coaching other professionals. Acquiring these skills will mean expanding in situ clinical training components. Providing the necessary venues will require the allocation of targeted resources.

#### Research

Health care research should be expanded to find reliable and ambulatory quality indicators to adequately evaluate new health care models [1]. This study can provide a foundation for further research on the effectiveness and optimization of care models including APNs. Possible areas for future research include the ongoing assessment of APN-inclusive care models currently under development and a cohort study comparing residential residential long-term care facilities with and without APNs. Furthermore, the points of view of patients, family care givers, GPs and collaborating professional health personnel are there to be explored.

## Conclusion

This study extended the understanding of APNs’ everyday practices in Switzerland’s primary medical health care system. Our findings indicate that everyday core activities include providing extended clinical practice and serving as an on-site specialist for patients, their relatives and other care professionals. Surrounding this level, net activities create the web of interactions that hold the core activities together. At the outermost level, the cohesive activities help not only to develop and anchor these roles but also to develop the Swiss primary care system. Working within this care model, these results suggest that APNs can contribute substantially to successful co-management of patients with chronic diseases in primary care systems. They also suggest that the evaluation of such care models should move beyond assessing APN outcomes alone and look comprehensively at the effects of care models where co-management in interdisciplinary teams is practiced.

## Supplementary Information

**Additional file 1. **Codetree.

## Data Availability

The datasets generated and/or analyzed during the current study are not publicly available due to confidentiality issues (given that the setting of the study is known and the limited number of participants, data is traceable) but are available from the corresponding author on reasonable request.

## Abbreviations

APN: Advanced Practice Nurse
BMI: Body mass index
CNS: Clinical Nurse Specialist
ECTS: European Credit Transfer System
GP: General Practitioner
NP: Nurse Practitioner
NYHA: New York Heart Association
OECD: Organization for Economic Collaboration and Development
TA: Thematic Analysis
US: United States
