# Supplementary figures and images for: Daily practices of advanced practice nurses within a multi-professional primary care practice in Switzerland: a qualitative analysis
Source: BMC Prim Care. 2023 Jan 21;24:26. doi: 10.1186/s12875-023-01977-y (PMC9862513; doi:10.1186/s12875-023-01977-y)

## Supplementary File 1

Code tree

**
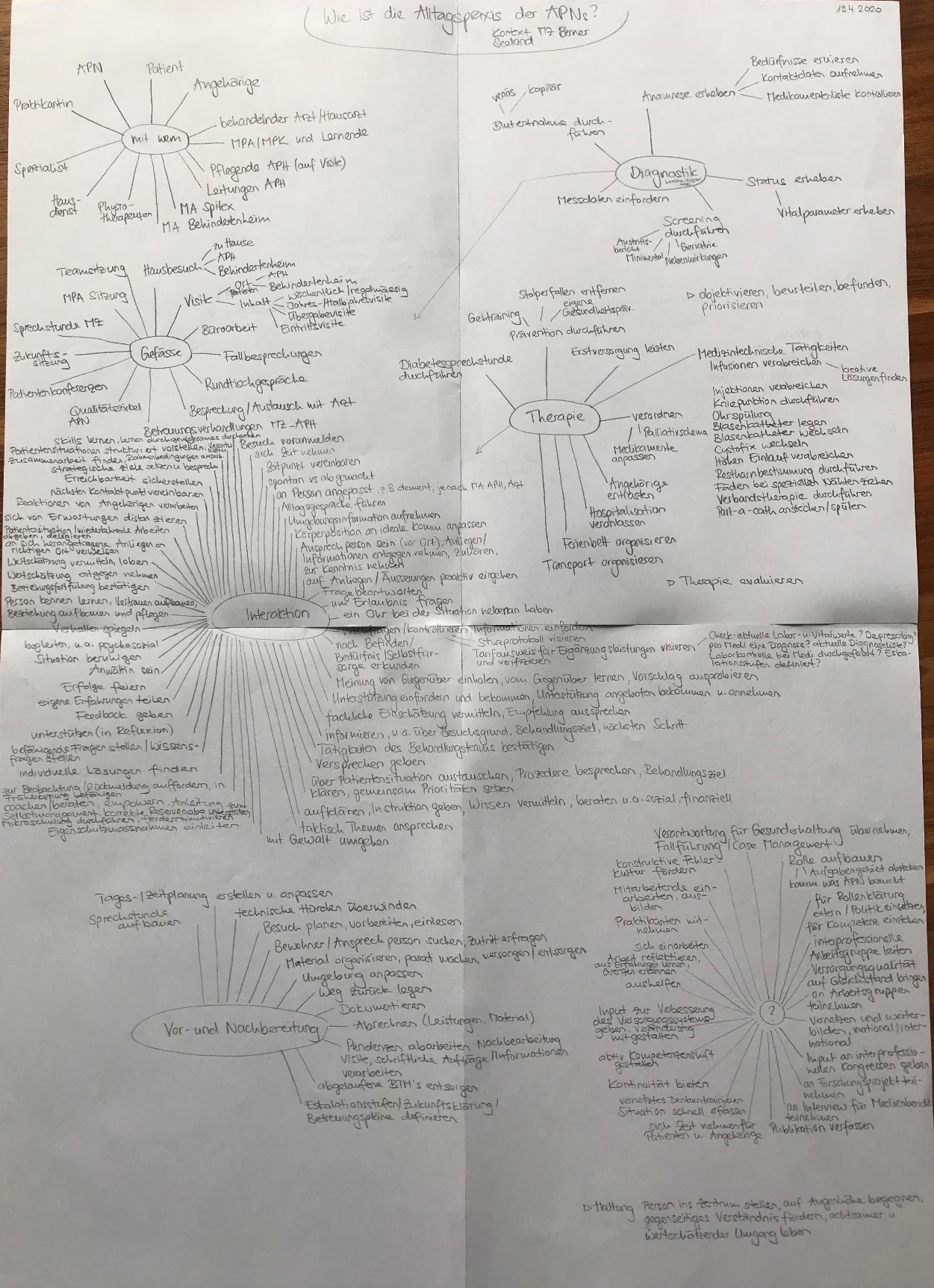
**

Supplement: Supplementary file 1 — Additional file 1. Codetree. [file 12875_2023_1977_MOESM1_ESM.docx]
